# Tracheobronchopathia osteochondroplastica—stalactite of airways

**DOI:** 10.1002/rcr2.790

**Published:** 2021-05-27

**Authors:** Suat Yee Lim, Mohd Faizul Abu Samah, Lalitha Pereirasamy, Bee See Chew, Irfhan Ali Hyder Ali

**Affiliations:** ^1^ Department of Respiratory Medicine Penang General Hospital Penang Malaysia; ^2^ Department of Pathology Penang General Hospital Penang Malaysia

**Keywords:** Chronic cough, tracheobronchial disorder, tracheobronchopathia osteochondroplastica

## Abstract

A case of tracheobronchopathia osteochondroplastica (TO) was diagnosed in a 53‐year‐old man, who presented with prolonged cough and recurrent respiratory tract infection. Bronchoscopy revealed exostosis over the anterolateral wall of trachea and main bronchi sparing the posterior membranous wall. The endobronchial biopsy subsequently revealed ossification of the cartilage. To date, the aetiology of this condition remains unknown, and treatment is mainly symptomatic, emphasizing on timely management of recurrent respiratory infections. Bronchoscopy or surgical intervention is usually reserved for symptomatic patients with severe airway narrowing and airflow obstruction.

## Graphical abstract

Tracheobronchopathia osteochondroplastica (TO) is a rare benign disorder affecting the trachea and main bronchi characterized by the development of submucosal cartilaginous and bony nodules. We report a case of TO in a patient presented with prolonged cough and recurrent respiratory tract infection.

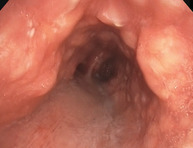

## Introduction

Tracheobronchopathia osteochondroplastica (TO) is a rare benign disorder affecting the trachea and main bronchi. It is characterized by the development of submucosal cartilaginous and bony nodules. These features are typically seen in computed tomography (CT) of the thorax and flexible bronchoscopy, whereas biopsy and histological identification is essential in making the diagnosis. First described by Rokitansky, Luschka, and Wilks in the 19th century [1, 2, 3, 4], TO was initially discovered in autopsy before the development of bronchoscopy and thoracic imaging leading to more reported cases [5]. Due to its rare occurrence, recognizing TO is essential as this affects its subsequent management. Here, we present a case of TO in a patient with chronic cough and recurrent lower respiratory tract infection.

## Case Report

A 53‐year‐old man presented with chronic productive cough with greenish sputum for the past six months. Further history revealed intermittent fever with no symptom of breathlessness or haemoptysis. He worked as a construction worker for the past 20 years. Otherwise, he had no significant medical illness or history of tobacco use. No family history of lung disease or malignancy was reported as well. Prior to this, he had been treated with multiple courses of antibiotic therapy without clinical improvement.

**Figure 1:**
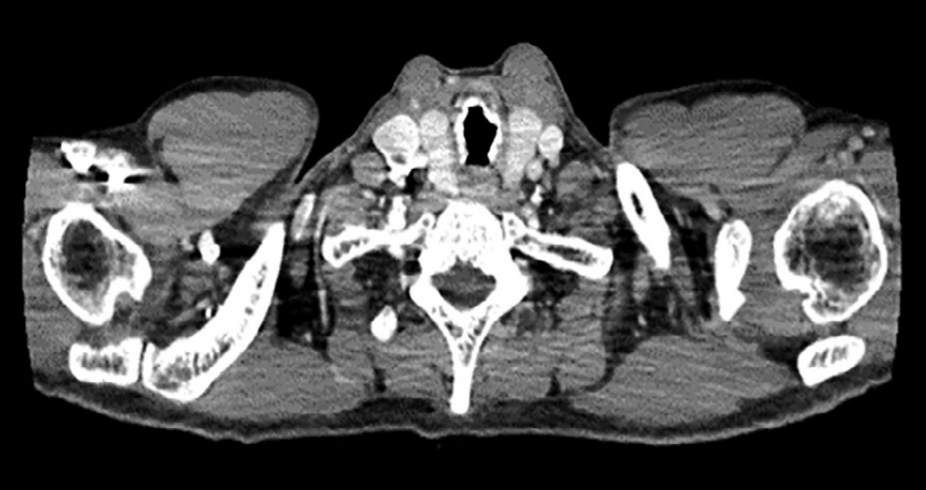
Computed tomography (CT) of the thorax showed variable degrees of stenosis and nodular calcified excrescences were seen protruding into the lumen of trachea.

**Figure 2:**
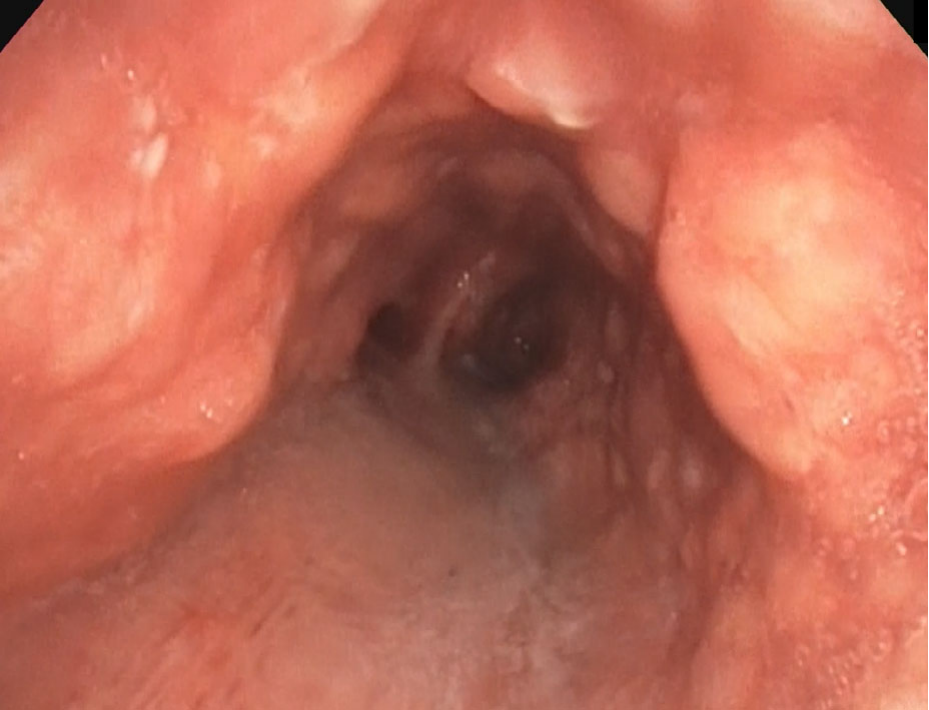
Flexible bronchoscopy showed dozens of white nodules (arrows) extruding into the lumen along the anterolateral wall of the trachea, sparing the posterior wall.

**Figure 3:**
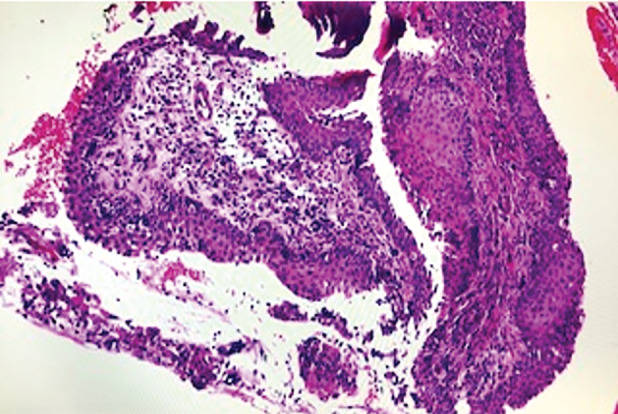
Prominent squamous metaplasia of the respiratory epithelium and chronic inflammatory infiltrates in the underlying stroma (200×, haematoxylin and eosin (H&E)).

**Figure 4:**
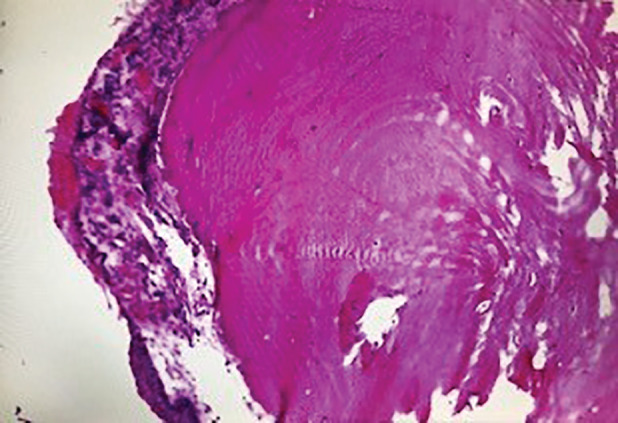
Pseudostratified columnar to stratified squamous epithelium with underlying subepithelial ossified nodule (400×, haematoxylin and eosin (H&E)).

Both systemic and chest examinations revealed no significant abnormalities and there was no lymphadenopathy. Further investigations with chest radiograph showed right middle zone consolidation and computed tomography (CT) of the thorax revealed subsegmental collapse with bronchiectasis of the right middle lobe and nodular calcified excrescences were seen protruding into the lumen of trachea and main bronchi (Fig. 1). There was no contrast‐enhancing lung mass or effusion. Flexible bronchoscopy showed patent, narrowed airway with nodular whitish granular excrescences along the anterolateral wall of the trachea and main bronchi, sparing the posterior wall as shown in Figure 2. Histopathological examination of the endobronchial biopsy revealed pseudostratified columnar to metaplastic squamous epithelium with underlying subepithelial nodules of osseous element composed of a central island of osteocytes (Figs. 3, 4). Other investigations including bronchial washing excluded pulmonary tuberculosis and concurrent infections, while spirometry revealed features of intrathoracic upper airway obstruction and routine blood investigations were normal. The diagnosis of TO was made after considering the radiological, bronchoscopic, and histopathological findings. He was treated with a course of intravenous antibiotic and subsequently discharged well and scheduled for follow‐up and bronchoscopy surveillance.

## Discussion

TO is an uncommon benign large airway disorder characterized by bony or cartilaginous nodules on tracheal and bronchial wall. With only less than 400 cases of TO reported in the literature [5], it is more commonly seen in patients between fourth and seventh decades of life [4, 6]. Bronchoscopy remains the gold standard in diagnosing this condition with direct visualization and reveals a characteristic cartilaginous nodule on the anterolateral part of trachea sparing the posterior membranous wall [4, 5]. Biopsy can be difficult due to the hard nature of the nodule. Despite that, a successful sampling of the lesion helps in confirming the diagnosis, and excludes other differential diagnosis such as bronchogenic carcinoma, tracheal amyloidosis, papillomatosis, and sarcoidosis [4, 6]. Histopathological findings include epithelial squamous metaplasia, submucosal cartilage, and ossification [5].

TO often follows a benign course [4] where patient commonly presents with symptoms such as chronic productive cough, haemoptysis, dyspnoea, wheezing, or recurrent pulmonary infection. The clinical presentation of TO is variable and ranges from incidental diagnosis in asymptomatic patients to severe disease with central airway obstruction. Patients are often misdiagnosed as asthma due to the symptoms of wheezing [7]. Our patient presented with right middle lobe syndrome due to obstructing submucosal lesions. Aetiology of TO remains unknown, although multiple theories such as airway inflammation, ecchondrosis, and exostosis arising from the cartilaginous tracheal rings and metaplastic changes of submucosal tissue were postulated to be contributing to the formation of the nodules [6]. To date, treatment mainly focuses on treating infectious complication, and only in cases of severe stenosis, bronchoscopic removal of the obstructing lesion will be indicated.

In summary, TO should be considered as a possible differential diagnosis in patients presenting with chronic cough and recurrent respiratory infection. Albeit rare, early recognition of this rare disorder is important in guiding appropriate management for these patients.

### Disclosure Statement

Appropriate written informed consent was obtained for publication of this case report and accompanying images.

### Author Contribution Statement

Suat Yee Lim and Mohd Faizul Abu Samah conceived the idea and wrote the manuscript with consultation and guidance of Lalitha Pereirasamy and Irfhan Ali Hyder Ali. Bee See Chew contributed to the analysis and description of the histological image. All authors reviewed and gave the final approval of the version to be published.
